# Lest we forget: Dr Michael Ellis DeBakey (1908–2008)

**DOI:** 10.1177/09677720231198505

**Published:** 2023-11-10

**Authors:** Hareesha Rishab Bharadwaj, Mahnoor Javed

**Affiliations:** 1 Faculty of Biology Medicine and Health, 5292 The University of Manchester, Manchester, UK; 2 School of Medicine, The University of Nottingham, Nottingham, UK

Modern History has witnessed many individuals who have been at the forefront of innovation and research. Amongst these, a vast multitude of luminaries stands Dr Michael Ellis DeBakey, whose innovative thought process and skilled hands saved the lives of many during his day. Over the course of his long and illustrious career, Michael contributed immensely to medicine and surgery; from developing the first artificial heart, creating innovative surgical procedures, and assisting with the American War Effort to establishing a world-class medical centre in Houston, Texas. Despite this, his stern demeanour, and the high expectations he possessed of his colleagues did not fail to attract criticism. To this end, this piece aims to narrate and provide an overview of his story (Figure 1).

## Humble beginnings

Michael Ellis DeBakey was born on the 7th of September, 1908 in Lake Charles, Louisiana. Born to a family of immigrants from then-called Ottoman Syria (now Lebanon), young Michael DeBakey was the oldest of five children. His parents, Shaker Morris and Raheeja DeBakey, had immigrated to pursue the American dream; Shaker was an established businessman in the field of pharmacy and healthcare, whereas Raheeja was a seamstress. Even as an established surgeon, DeBakey often referred to his childhood with a distinct sense of fondness and fascination; his parents, who were his greatest inspiration, had instilled a sense of curiosity and inquisitiveness in all their children.

During an interview with William Roberts in 1996, Michael said, ‘First, Bill, I was blessed with parents who were both highly intelligent and exceedingly kind and generous in their temperament and psyche. They lived almost exclusively for their children. They wanted to give us the best of everything, and they believed education was crucial’.^2^

Young Michael DeBakey was curious, and prying and would often indulge in a plethora of boyhood endeavours, from fixing car engines, gardening, and playing the saxophone, to sewing, which his mother taught him.^3^ DeBakey was also remarkably excellent at school; he was regarded to be extremely studious, and at one point of time, was even permitted to skip a grade because of his academic rigour.^4,5^ In addition, he would often accompany his father to business meetings, and it was during these meetings that he came across physicians affiliated with his father's business. These interactions and discussions birthed his love for medicine.^5^

## Education, training and travelling across the pond

Michael DeBakey enrolled into a combined undergraduate bachelor of science and graduate medicine degree at Tulane University, graduating with a BSc in 1930 and MD in 1932. Despite the rigorous academic demands of his course, DeBakey still explored his curiosity to the fullest and had not let his curiosity and inquisitiveness from childhood wane. He worked in surgical research whilst in medical school under the supervision of Rudolph Atas and Alton Ochsner.^6^

The relationship between DeBakey and Alton Oschner was a strong one, based on mutual admiration and respect for one another. During his sophomore year, DeBakey worked as an assistant technician for a member of the Tulane University Medical Faculty. It was during this time that he was approached by Dr Ochsner, and this would prove to be the foundation of one of the most significant relationships and partnerships in his life.^2,5^

It was during this time, when he was working in Dr Ochsner's lab, that DeBakey innovated the development of a primitive version of the roller pump (he had combined old pumps and rubber tubing to develop the device). This device, initially developed with the intention of helping in blood transfusion, would then go on to become an essential component of the heart-lung machine.^6^

‘In the early nineteenth century, someone had written an article about the use of rubber tubing (rubber had just been discovered), and about compressing that tubing to force fluid out of it as a possible pump. That is what gave me the idea. So I began to think about a way to make a rubber tube into a pump. I got the idea of some kind of compression. I worked on different ways of compressing, pressing it down, finally rolling something over it as I could with my fingers. I experimented with different ways of doing this. I went to a foundry in New Orleans, and had them make a kind of round cup that I could put the tube in. Then I experimented with different types of rollers, 2 rollers, 3 rollers, 4 rollers. Finally, after a year or 2 years of experimenting, I finally got the 2 rollers and the pump the way I wanted it’.^2^

When DeBakey had completed medical school, Dr Ochsner told him, ‘I want you to stay in surgery. You impress me. I want you to be my intern and resident’. So I became, from that point on, his student, and he became my mentor. He treated me like a son. We wrote papers together. I would go over to his house and work on a paper in his study at his home and make slides for him. We had to do everything ourselves in those days. It was a great learning experience, and he was a great disciplinarian’.^2^

DeBakey spent a year as an intern at the Charity Hospital in New Orleans, followed by a residency in surgery at the University of Michigan. In 1936, he travelled to Europe to study under several leading surgeons, including Martin Kirschner in Germany and René Leriche in France. It was Dr Ochsner's personal recommendation that Michael completes his postgraduate training in Europe, being of Swiss-German heritage himself.^5^ Michael DeBakey often referred to his days in Europe as being the catalyst in his pursuit and conquest of knowledge; in Europe, he was not only involved in the pursuit of surgical training but also had to learn the local language – which was one thing he particularly did not mind. He initially travelled to France, to work with Prof. René Leriche – who was considered to be one of the most advanced vascular surgeons at the time. In the interview with William Roberts, Michael quotes:

He was more philosophical, introspective, a great historian, and well versed in art. He was one of those old-fashioned, well-educated Frenchmen, and surgery was almost an avocation. He was interested in the circulation, and if you read some of his articles, you can see how philosophical he was. He wrote beautifully.^2^

He later travelled to Germany to work with Prof Martin Kirschner to learn from his expertise in Gastroenterology. The two fostered a cordial bond, extending beyond work, to friendship and compassion – something that was quite seemingly rare between the Germans and the Americans at the time, owing to political stresses. On Prof Martin Kirschner, Michael quotes:

He was a very good surgeon. He was very kind to me; at least once a month he would invite me to his home for dinner. His wife was also nice, and so was his daughter.

It was also during this time that DeBakey began to develop an interest in cardiovascular surgery, a field that was still in its infancy. He subsequently returned to the United States.^2^

## War, the military, and the birth of the national library of medicine

Displaying tremendous talent, and receiving excellent mentorship, Michael DeBakey was well on his path to becoming a renowned surgeon. Unfortunately, it was also during this very time that World War II broke out; DeBakey willingly volunteered to participate in the war effort, despite the prospect of being declared ‘essential’ and hence exempted from conscription.^6^ Recognising his talent and rigorous pursuit, he was recruited into the Surgeon General's Office by Fred Rankin.

When Fred Rankin, who was the Chief Surgical Consultant in the Surgeon General's Office of the Pentagon, learned that I wanted to go into the service, he told Dr Ochsner that he wanted me in his office.^2^

During his time working for the Surgeon General's office, DeBakey advocated heavily for the devolution of the Surgeon General's National Library of Medicine from the U.S. army. Whilst working there, he noticed its value and its potential of becoming a treasure of medical literature and also particularly noticed the poor state it was in. At the time, given the war effort, the army was reluctant to utilise funds to revamp the library, and Michael's request did not proceed further.^4^

When it rained, they had to put tarpaulins over the books. They had a commode outside the building. The stacks were so crowded, in fact, that I wrote an article called ‘Chaos Among the Stacks’. I started making noises about that while I was still in the service. The Surgeon General said, ‘You know, Mike, we have been trying for 30 years to get a new building. We’ve put in a request every year; we just can’t compete with tanks.’ That convinced me that the Library did not belong in the Army.^2^

Following the War, DeBakey took it upon himself to advocate for this change and utilised his influential connections in politics to help him with this. With the help of Senator Hill, and with the backing of many influential politicians, this bill would be approved by the U.S. Congress in 1956.

Today, the National Library of Medicine, headquartered in Bethesda, Maryland, is home to millions of articles and has become a repository of medical knowledge.

Besides this, DeBakey was also heavily involved in medical administration. He is credited for the development of Mobile Army Surgical Hospital units; a concept based on the ideology that soldiers would be better treated in the forefront of war, rather than being transported many miles away to a single centre. This innovation was responsible for saving many, many Allied Soldiers’ lives. President Truman also commissioned Michael to revamp the Navy Hospital – an institution that focused on the long-term care of U.S. army veterans. This today is known as the Veterans Administration and the Veterans Affairs Medical Centres (Figure 2).^4^

## Criticism

Notwithstanding the myriad contributions rendered by Michael DeBakey, a subset of his peers harboured substantial reservations and expressed critique regarding his work ethic. Despite his adeptness as an educator and a proponent of progressive medical pedagogy, DeBakey's patience waned when faced with colleagues unable to grasp complex concepts. He consistently held elevated expectations from his counterparts, frequently extending beyond conventional attainability, and occasionally exhibited disdain for those unable to generate and articulate innovative insights across domains encompassing surgery, governmental policies, and broader life affairs.^8^ Interrogated concerning the origins of his exacting standards during a 1972 interview, DeBakey responded, embodying an unwavering commitment to excellence and insatiable pursuit of intellectual prowess,

That's the whole definition of mediocre. It's very difficult for me to put up with mediocrity. The most difficult thing I have to deal with is to put up with mediocrity. I still can't get adjusted to it, and what happens is that I'm turned off to these people, I don’t want to associate with them. I don't want to teach them; I don't do anything to them. I just want to leave them alone, just not worth fooling with.^9^

Michael DeBakey's mastery as a surgeon extended to a meticulous focus on formal documentation of his groundbreaking surgical techniques, aimed at advancing surgical education. However, this laudable approach was not without criticism, notably centred around concerns over patient privacy and consent. DeBakey's inclination to record surgeries, while a valuable educational tool, raised ethical eyebrows within his professional circle.^10^ Furthermore, within the realm of surgical education, his demeanour earned him a reputation as a demanding and stern taskmaster in the operating theatre. His stringent discipline expectations for surgical trainees and establishment of a hierarchical structure met with disapproval from some quarters, particularly those who found themselves at odds with this strict framework.^8^

Reflecting his steadfast pursuit of excellence, DeBakey exhibited a degree of intolerance towards those perceived as hindrances to his aim, instilling a sense of contempt for those who failed to align with his unwavering commitment to perfection. Beyond the realm of surgical education, DeBakey's practice of ‘overlapping surgeries’ drew scrutiny. This approach, involving his sequential involvement in multiple surgeries, attending only to critical phases and delegating the rest, drew criticism from colleagues who questioned the optimal allocation of surgical responsibilities.^8^ In a resolute pursuit of professional excellence, DeBakey's multifaceted legacy encompassed both groundbreaking surgical contributions and intricate interpersonal dynamics that spurred discussions and critique among his contemporaries.

## Post war: Innovation and research

Following his extended duration in the Army, Michael DeBakey, now almost 40, returned to a full-time medical career. During this period, he made several important contributions to the field of vascular surgery, including the development of new techniques for repairing aneurysms and bypassing blocked arteries. He also became interested in the use of polyethylene terephthalate (Dacron) for repairing blood vessels, which would later become a major focus of his research.^11^

In the 1950s, DeBakey began to focus on cardiovascular surgery, which was still a relatively new field at the time. He made several ground-breaking contributions during this period, including the development of new surgical techniques for repairing and replacing damaged heart valves, as well as the first successful implantation of a ventricular assist device in a human patient. During the final decades of his career, DeBakey became increasingly involved in research and innovation, and he continued to make major contributions to the field of cardiovascular surgery. In 1963, he co-founded the Texas Heart Institute in Houston, which quickly became one of the world's leading centres for cardiovascular research and treatment. He also worked on the development of the first artificial heart, which was successfully implanted in a patient in 1966. Although the patient survived for only a few days, the procedure represented a major milestone in the field of cardiac surgery.^12–18^

DeBakey's contributions to the field of medicine have been recognised with numerous honours and awards throughout his career. In 1969, he was awarded the Presidential Medal of Freedom, the highest civilian honour in the United States. He also received the National Medal of Science, the Lasker-DeBakey Clinical Medical Research Award eponymously named and numerous other accolades. In addition, he was a member of the National Academy of Sciences and the National Academy of Medicine. Following his death, he was granted ground burial at Arlington National Cemetery by the Secretary of the Army.^14^

**Figure 1.**
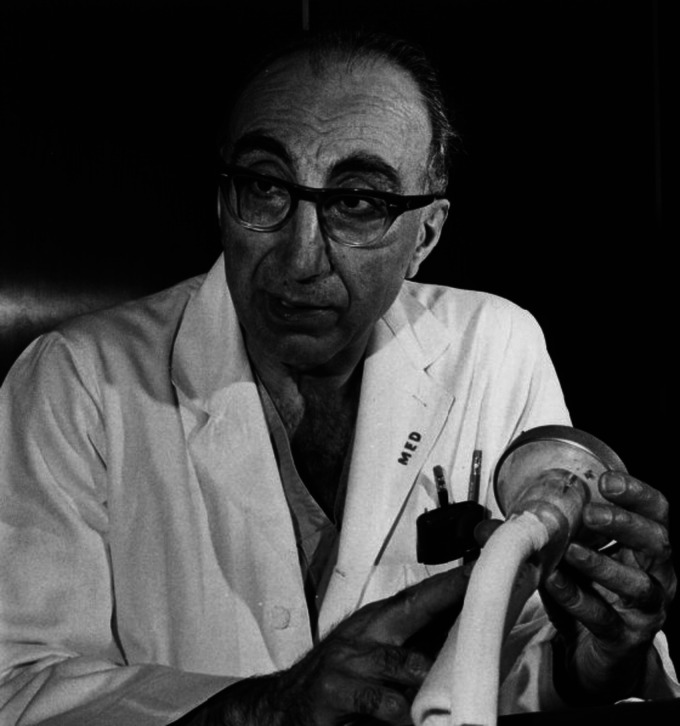
Dr Michael Ellis DeBakey. Image Courtesy of Special Collections, University of Houston Libraries.^1^

**Figure 2.**
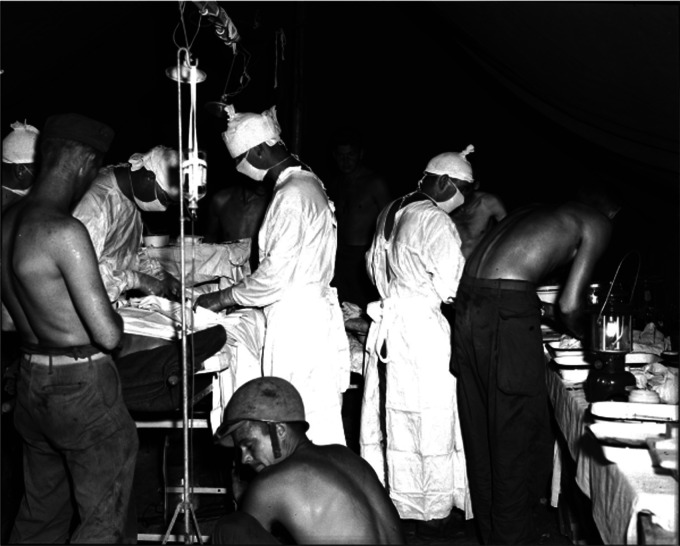
A Mobile Army Surgical Unit operating to treat wounded American soldiers. Image Courtesy of the National Museum of Health and Medicine.^7^
